# Editorial: Data mining methods for analyzing cognitive and affective disorders based on multimodal omics, volume II

**DOI:** 10.3389/fpsyt.2023.1273351

**Published:** 2023-11-24

**Authors:** Tao Wang, Elaine Stella Chapman, Zhongyu Wei, Jiajie Peng

**Affiliations:** ^1^School of Computer Science, Northwestern Polytechnical University, Xi'An, China; ^2^Key Laboratory of Big Data Storage and Management, Ministry of Industry and Information Technology, Northwestern Polytechnical University, Xi'An, China; ^3^University of Western Australia, Perth, WA, Australia; ^4^School of Data Science, Fudan University, Shanghai, China

**Keywords:** cognitive and affective disorders, data mining methods, deep learning, multi-modal data, omics

Cognitive disorders, such as amnesia, dementia, and delirium, form a category of psychiatric disorders that primarily impede learning, memory, perception, and problem-solving. Simultaneously, affective disorders, encompassing conditions like depression and bipolar disorder, represent another set of psychiatric disorders. While these categories differ, they may present overlapping symptoms and can even intertwine. For instance, recent research indicates that affective issues, such as depression, could potentially heighten the risk of developing dementia. Although there have been significant advances in the realm of knowledge discovery regarding both cognitive and affective disorders, the challenge of understanding the interaction between these two categories still persists.

Fortunately, the rapid evolution of experimental technologies has granted researchers the ability to acquire multi-modal or multi-omics data in individual studies. When investigating cognitive and affective disorders, a variety of data types may be utilized, including Electroencephalography (EEG), functional Magnetic Resonance Imaging (fMRI), genomics, phenomics, transcriptomics, radiomics, and single-cell omics. Additionally, machine learning applied to brain MRI data plays a vital role in identifying biomarkers and patterns associated with cognitive and affective disorders, facilitating early diagnosis and targeted treatments (1). To extract valuable insights from multi-modal omics data, data mining techniques such as statistical methods, unsupervised learning, supervised learning, and network-based methods have gained popularity. These data and methods provide opportunities for knowledge discovery in the field of cognitive and affective disorders, particularly in understanding the interaction between the two. Therefore, here we present a continuous Research Topic on “*Data mining methods for analyzing cognitive and affective disorders based on multimodal omics, volume II*” followed by the inaugural issue.

This Research Topic aims to provide a platform for researchers across diverse fields to share advanced data mining methods based on multi-modal omics. This will enrich our comprehension of cognitive and affective disorders, with a particular focus on unraveling the intricate relationships between these two types of disorders. After rigorous peer-review, a total of five outstanding articles were selected for this topic collection. Below we highlighted these articles.

Liang et al. takes a comprehensive look at the correlation between homocysteine levels and poststroke depression (PSD). Drawing upon a broad selection of studies, involving a total of 2,907 patients, the meta-analysis aimed to shed light on the conflicting findings of previous research on this topic. The results were striking. Liang et al. found that the top vs. bottom homocysteine levels correlated to a pooled adjusted odds ratio (OR) of PSD of 3.72, suggesting that elevated homocysteine levels at the acute stage of an ischemic stroke might serve as an independent predictor of PSD. Moreover, the predictive value was found to be stronger in follow-up periods of 6 months or more. Further, a per unit increase in homocysteine level was found to raise the risk of PSD by 7%.

Liu et al. proposed a novel, AI-based approach to assessing the severity of depression in real-time. Recognizing the limitations of traditional diagnostic methods such as psychological scales and doctor-patient interviews, the authors developed a multi-modal deep convolutional neural network (CNN) model, which could extract patients' facial expressions and body movements from video footage. They established the behavioral depression degree (BDD) metrics, a new measure of depression severity. Their findings show a high correlation (over 74%) between BDD and established measures such as the self-rating depression scale (SDS), self-rating anxiety scale (SAS), and Hamilton depression scale (HAMD). Moreover, this work found that BDD could effectively track the changes in patients' depression states over different stages of treatment.

Chen et al. presented a promising approach to improving the accuracy of emotion recognition using EEG data. Recognizing that emotions play a significant role in human-computer interaction, Chen et al. proposed a feature extraction method that capitalizes on the temporal information present in EEG signals. They introduced the concepts of “microstates” and “momentary quasi-stable scalp electric potential topographies”, and combined them with the k-mer method, a computational technique widely used in genomics and sequence analysis. The application of these techniques allows them to extract fine and coarse-level features, which when combined, dramatically improve the classification accuracy of the emotion recognition process.

Song and Zhao utilized deep neural networks to explore the complex dynamics of courtroom emotions. Recognizing the dearth of research on how sound influences emotions in court, Song and Zhao developed a predictive model using convolutional neural networks and long short-term memory networks to analyze speech signals and infer emotions. Analyzing voice data from fifty trial records, Song and Zhao found that the judge's voice significantly influences the defendant's emotions, with expressions of anger often triggering fear in the defendant. This study provides a unique and valuable insight into the emotional dynamics of the courtroom environment.

Zhao et al. addressed a crucial gap in our understanding of how metabolites are related to human diseases. Utilizing multi-source heterogeneous microbiome data, Zhao et al. proposed a novel algorithm, the logical matrix factorization and local nearest neighbor constraints (LMFLNC) method, to predict metabolite-disease interactions. By creating metabolite-metabolite and disease-disease similarity networks, the method was able to calculate the probability of metabolite-disease interactions based on the learned latent representations of metabolites and diseases. The LMFLNC method outperformed other existing algorithms, suggesting its effectiveness in predicting metabolite-disease interactions, and thereby improving disease diagnosis and treatment.

At least two professional domain experts are required during the peer-review process. We would like to express our sincere appreciation to all authors for their invaluable contributions, and our deep gratitude toward our panel of reviewers, whose expertise and critical insights have ensured the quality and scientific rigor of the published work. We believe that the knowledge shared and generated within this issue will stimulate further innovation and foster continued dialogue in this critical field.

## Author contributions

TW: Conceptualization, Writing—original draft, Writing—review & editing. EC: Conceptualization, Writing—review & editing. ZW: Conceptualization, Writing—review & editing. JP: Conceptualization, Writing—review & editing.
